# Ancient Schwannoma of the Cauda Equina: Our Experience and Review of the Literature

**DOI:** 10.1155/2016/7930521

**Published:** 2016-12-22

**Authors:** Venceslao Wierzbicki, Alessandro Pesce, Luigi Marrocco, Emanuele Piccione, Alessandro Frati, Riccardo Caruso

**Affiliations:** ^1^Neurosurgery Department, Army Hospital of Rome “Celio”, Roma, Italy; ^2^NESMOS Department, “Sapienza” University, Roma, Italy; ^3^Neurosurgery Department, Azienda Ospedaliera Sant'Andrea, Roma, Italy; ^4^Neurosurgery Division, IRCCS “Neuromed”, Pozzilli, Italy; ^5^Neurology and Psychiatry Department, “Sapienza” University, Roma, Italy

## Abstract

Ancient schwannomas (AS) are exceedingly rare variant of common schwannomas (CS). Only two cases involving the cauda equina region have been previously reported in literature. AS are typically associated with a higher histological degree of degenerative changes (Antoni B areas). It is of peculiar importance, according to our opinion, to outline that, because of their extremely slow growth (which explains the increase of the degenerative changes in respect to the CS) and their typical soft consistency in respect to their standard counterparts, AS usually imply an even better prognosis.

## 1. Introduction

To the best of our knowledge, there are only 2 cases previously reported of AS involving cauda equina [1, 2], an exceedingly rare schwannoma subtype for an extremely uncommon location. Although reported to affect head and neck, cervical region, limbs, pelvis, and retroperitoneum [1–5], it is uncommon is the spinal cord [6–10] but cauda equina involvement is exceptional.

The authors report a case of AS involving cauda equina, an exceptionally rare lesion for an extremely rare location, and review all the relevant literature in order to outline clinical, radiological, and pathological remarks of this rare condition.

## 2. Case Presentation

A 36-year-old man was admitted to our department complaining of one-year history of progressively worsening back pain with bilateral radiation to the anterior surface of the lower limbs, combined with paresthesias to both lower limbs and urinary incontinence for about a month. The neurological examination reported a bilateral fall of both lower limbs in Mingazzini maneuver and Patellar and Achilles hyporeflexia and bilateral sensory deficit of sacral dermatomes.

**Figure 1:**
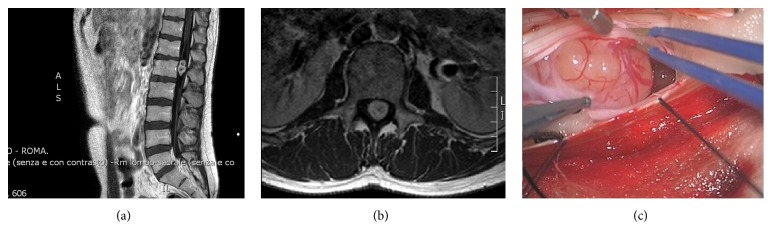
(a) Sagittal and (b) axial preoperative contrast enhanced MRI scan. (c) Intraoperative image of the lesion at gross examination resembles an ordinary schwannoma. Note the “target” pattern in (a) and the finely heterogenous contrast enhancement in (b), consistent with the typical aspect of an ancient schwannoma.

The patient underwent a lumbar spine MRI scan with TSE, FS, T1w, and T2w sequences and contrast enhanced T1 (Gadolinium) which demonstrated a voluminous intradural-extramedullary tumor (1.9 cm ×  2.8 cm ×  2.0 cm), with solid and cystic areas of heterogeneous intensity signal (Figures 1(a) and 1(b)) with a strong Gadolinium enhancement.

The patient was operated on for D12-L1 laminotomy and en bloc resection of the lesion. Gross pathology was consistent with a spinal schwannoma (Figure 1(c)). The postoperative course was uneventful and the patient experienced a complete and immediate neurological recovery and was discharged in third postoperative day.

Histology showed a mesenchymal neoplasm, with an alternation of hypercellular (Antoni A) and hypocellular areas (Antoni B) presenting degenerative traits like multiple microcystic areas filled with a tenuously basophilic colloid material in H&E stain, surrounded by a loose hypocellular fibrillary stroma with rare necrotic areas.

**Figure 2:**
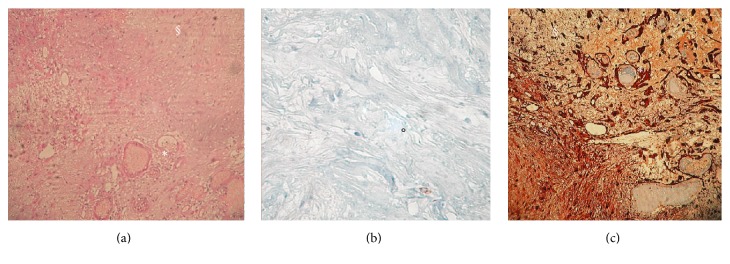
(a) H&E 40x, (b) Ki67, and (c) S-100 immunostaining of the lesion. Note the presence of microcystic areas (*∗*) in a loose fibrillary stroma (§) finely admixed with hypercellular areas composed of bundles of spindle-cells (°).

The neoplasm was composed of spindle-cells with pyknotic nuclei and rare mitotic figures and a modest degree of nuclear pleomorphism. The neoplasm showed a remarkable S-100 immunoreactivity and a scarce reactivity for GFAP staining. The Ki67 immunoreactivity demonstrated an extremely low proliferating rate (less than 1%) (Figures 2(a), 2(b), and 2(c)). Histology was consistent with a typical AS.

Before writing this paper patient was consulted and gave informed written explicit consent to this report.

## 3. Discussion

Ancient schwannoma is a rare variant of schwannoma (<1% of CS) [1]. It is a benign nerve sheath tumor originating from the Schwann cells covering the peripheral nerves [6]. Up to 20% of cases are associated with type 1 neurofibromatosis [11]. It is more common in female sex; and its peak of incidence lies between the second and fifth decades [12]. It mostly affects the head and neck, cervical region, pelvis, retroperitoneum, and flexor aspect of the limbs [1–5].

Cauda equina tumors are rare entities with a total incidence of less than 6% among spinal primary neoplasms [13]; CS count for about 50% of cases [14]. To the best of our knowledge, only two cases of AS have been previously reported [1, 2]. In the first case, the AS involved the conus medullaris-cauda equina region; and the patient complained of a long history of progressively worsening low back pain with minor neurological impairment. In the second case, the AS was completely intrasacral and involved sacral nerve roots [2]. The patient complained of lumbar pain. The cohort in which this female patient was included had an average duration of preoperative symptoms of 6.7 years (Table 1).

Another case of AS was reported by Hayashi et al. [9] that was located at the epiconus-conus medullaris, but it was purely intramedullary and therefore quite different for what concerns both clinical presentation and surgical implications.

These tumors are typically associated with a long lasting history of mild low back pain, progressively worsening over years [3, 15]. It is common for neoplasm involving the cauda equina to reach considerable dimensions before motor and sensory deficits appear [15, 16]. Neurological signs are related to the compressive effects of the lesion on the nerve roots [3].

Occasionally, the clinical onset may be a tumor hemorrhage [10, 17], located nearby or distant from the tumor site [18, 19]. It can rarely simulate a Charcot-Marie-Tooth syndrome [20] or an intracranial hypertension with a bilateral papilledema [21, 22]; however, in most cases, even a giant schwannoma located within the spinal canal can be totally asymptomatic [23].

As for any other primary spinal neoplasm, Gadolinium enhanced MRI is the current “gold standard” for radiological diagnosis [1, 3], and the histological features of the AS reflect MRI findings.

The radiological appearance of the capsule and of the central “core” of the lesion is important in differentiating a CS from its “ancient” variant [3, 5, 24]. The outer aspect of a CS, composed mostly of Antoni B regions, is hypointense in T1w and hyperintense on T2w. The core of the lesion is usually hypo/isointense, both on T1w and on T2w, with strong a Gadolinium enhancement which is typical of the Antoni A regions [3]. These features realize a “target” pattern which is present in up to 52% of benign peripheral nerve tumors [5].

In AS, Antoni A and B areas are more finely admixed; thus, the resulting MRI appearance is of a circumscribed, rounded mass with heterogeneous contrast enhancement [3, 5].

Because of the different pattern of contrast enhancement in respect to CS and the presence of multiple cystic areas, the differential diagnosis between AS and malignant neoplasms such as fibrous histiocytomas, malignant peripheral nerve sheath tumors, liposarcomas, or haemangiopericytomas can be quite difficult [3] before histological confirmation.

Macroscopically, the AS is not different from the standard schwannoma: but there are remarkable histological differences.

CS is a mesenchymal neoplasm composed of an alternation of bundles of spindle-shaped cells with pyknotic nuclei packed in pseudopalisades (Antoni A areas) and hypocellular areas with cells organized in ovular masses surrounded by a loose stroma (Antoni B areas); mitotic index is low but mitotic figures are commonly observed [1, 6, 7].

On the opposite, AS has a higher degree of degenerative phenomena involving Antoni B areas: microcysts, calcifications, intravasal thrombosis, necrosis, and hyalinosis are common findings. Nuclear pleomorphism is more pronounced than CS [1, 6, 25].

The aforementioned histological features are useful to perform the differential diagnosis between AS and mesenchymal malignant neoplasms. Furthermore the absence of local invasion involving the tumor capsule and neoplastic vessels and a low Ki67 immunoreactivity complete the diagnosis [6].

As for other intradural-extramedullary spinal neoplasms [26], surgery is mandatory to gain a safe and effective neurological relief and total oncological control of the disease [14]. A standard posterior midline approach to the thoracolumbar spine is the gold standard in most cases [26].

We strongly prefer laminotomy over laminectomy to approach an intradural-extramedullary lesion because of the risk of iatrogenic postoperative instability and spinal deformity. The incidence of postoperative instability in thoracolumbar spine can involve up to 25% of patients receiving two or more levels of laminectomies [27] and postoperative deformity affects 9.4% of patients undergoing laminectomy compared to 3% of laminotomy patients [28].

## 4. Conclusions

Ancient schwannomas are benign lesions characterized by a prevalence of Antoni B areas heterogeneously and finely admixed with Antoni A areas. It exceptionally involves cauda equina and only two other cases with cauda equina involvement have been previously reported. Surgical treatment is the gold standard for this condition.

## Figures and Tables

**Table 1:** The cases reported in detail.

Author	Age	Sex	Location	Symptoms	Duration	Therapy	Recovery
Saiful Azli et al. [1]	54	M	Conus medullaris-cauda equina	Worsening lumbar pain and sciatica	2 years	Surgical	Complete
Domínguez et al. [2]	39	F	Intrasacral, cauda equina involvement	Lumbar pain	Not reported	Surgical	Complete
Our case	36	M	Cauda equina	Lumbar pain and cruralgia	1 year	Surgical	Complete

## Abbreviations

AS: Ancient schwannoma
CS: Common schwannoma
MRI: Magnetic Resonance Imaging
T1w: T1 weighted MRI imaging
T2w: T2 weighted MRI imaging
TSE: Turbo spin echo
FS: Fat suppressed
H&E: Hematoxylin and Eosin
GFAP: Glial fibrillary acid protein.
